# Arterial endothelium creates a permissive niche for expansion of human cord blood hematopoietic stem and progenitor cells

**DOI:** 10.1186/s13287-020-01880-8

**Published:** 2020-08-14

**Authors:** Huilin Li, Haiyun Pei, Sihan Wang, Bowen Zhang, Zeng Fan, Yiming Liu, Xiaoyan Xie, Zhou Yang, Lei Xu, Yali Jia, Yun Bai, Yi Han, Lin Chen, Lijuan He, Xue Nan, Wen Yue, Xuetao Pei

**Affiliations:** 1 Stem Cell and Regenerative Medicine Lab, Institute of Health Service and Transfusion Medicine, Beijing, 100850 China; 2 Experimental Hematology and Biochemistry Lab, Beijing Institute of Radiation Medicine, Beijing, 100850 China; 3 South China Research Center for Stem Cell & Regenerative Medicine, SCIB, Guangzhou, 510005 China

**Keywords:** Arterial endothelial cells, Hematopoietic stem and progenitor cells, Expansion, Niche, Transplantation

## Abstract

**Background:**

Although cord blood (CB) offers promise for treatment of patients with high-risk hematological malignancies and immune disorders, the limited numbers of hematopoietic stem cell (HSC)/progenitor cell in a CB unit and straitened circumstances in expanding ex vivo make it quite challenging to develop the successful cell therapies.

**Methods:**

In this study, a novel strategy has been developed to support ex vivo expansion of hematopoietic stem and progenitor cells (HSPCs) by coculture with engineered human umbilical arterial endothelial cells (HuAECs-E4orf1-GFP), which expresses *E4ORF1* stably by using a retroviral system.

**Results:**

Coculture of CD34^+^ hCB cells with HuAECs-E4orf1-GFP resulted in generation of considerably more total nucleated cells, CD34^+^CD38^−^, and CD34^+^CD38^−^CD90^+^ HSPCs in comparison with that of cytokines alone or that of coculture with human umbilical vein endothelial cells (HuVECs) after 14-day amplification. The in vitro multilineage differentiation potential and in vivo repopulating capacity of the expanded hematopoietic cells cocultured with HuAECs-E4orf1-GFP were also markedly enhanced compared with the other two control groups. DLL4, a major determinant of arterial endothelial cell (EC) identity, was associated with CD34^+^ hCB cells amplified on HuAECs-E4orf1-GFP.

**Conclusions:**

Collectively, we demonstrated that HuAECs acted as a permissive niche in facilitating expansion of HSPCs. Our study further implicated that the crucial factors and related pathways presented in HuAECs may give a hint to maintain self-renewal of bona fide HSCs.

## Introduction

Hematopoietic stem cells (HSCs), resided at the apex of a complicated blood cellular hierarchy, can replenish themselves by self-renewal and give rise to all the other blood cells [1]. Currently, generation of HSCs from pluripotent stem cells (PSC), including induced pluripotent stem cells (iPSC) and embryonic stem cells (ESC), is probably out of reach [2–4]. And bone marrow (BM), umbilical cord blood (UCB), or mobilized peripheral blood (MPB) is the only source of HSCs presently available [5–7]. Consequently, establishing a system for ex vivo expansion of HSCs would open a unique opportunity to study human HSC self-renewal and provide a novel source of therapeutic cells for blood disorders. Achieving this goal requires a detailed understanding of crucial elements contributing to HSC amplification and function maintenance in hematopoietic niche in vivo.

Umbilical cord actually contains a number of mostly naïve HSCs until delivery [8]. However, the cellular basis of the niche contributing to HSC maintenance in the umbilical cord is not yet clearly elucidated. As for anatomic structure, apart from vascular smooth muscle cells and perivascular mesenchyme, hematopoietic cells are in close vicinity of endothelial cells (ECs). In fact, during mammalian embryogenesis, endothelial and hematopoietic cells develop in parallel [9]. Recent reports suggested that HSCs derive from hemogenic endothelial cells lining the aortic floor both in murine and zebrafish [10, 11]. Consistently, definitive hematopoiesis cannot occur in the absence of endothelial cell development and arterial specification in the embryo [12]. This research collectively indicated that the appearance of cells having definitive HSC characteristics is in close association with arterial endothelium. Notably, umbilical arteries have been identified by a number of investigators as one of the primary birthplace of HSCs except the dorsal aorta in the aorta-gonad-mesonephros (AGM) region and the vitelline arteries [13, 14], raising the possibility that arterial endothelial cells (AECs) within umbilical cord might harbor a complex network of signals to modulate the nascent HSCs. Interestingly, there is growing evidence that endothelium not only plays a fundamental role in the generation of definitive HSCs but also is essential for the self-renewal of HSCs in vitro or BM repopulation in vivo recently [15, 16]. These observations raise the question of whether AECs within the umbilical cord are also one of the niche components for HSC amplification and function maintenance. To investigate this possibility, we thus presumably speculated that an ex vivo human umbilical arterial endothelial cell (HuAEC) vascular niche would provide intrinsic regulators contributing to the expansion of HSCs.

Primary AECs have limited expansion capability and undergo de-differentiation in culture [17, 18], making physiologic application as hematopoiesis niche challenging. On this account, here we developed stable HuAEC lines possessed the conventional characteristics of AECs by transduction of *E4ORF1* and the *green fluorescent protein (GFP)* using retrovirus vectors (HuAECs-E4orf1-GFP). These based on the theory of E4orf1 as a “pro-life” signal to promote survival of primary endothelial cells (PECs) [19, 20]. Then we revealed that HuAECs-E4orf1-GFP have potentiality to create a permissive niche for expansion of hCB CD34^+^ cells, as determined by a conventionally defined set of markers for human hematopoietic stem and progenitor cells (HSPCs), colony assays, and in vivo repopulating capacity in NOD.Cg-Prkdc^scid^Il2rg^tm1Wjl^/SzJ (NSG) mice. Furthermore, we found that Notch signaling molecules contribute to the supportive effect of HuAECs-E4orf1-GFP. Our data show, for the first time, a functional link between HuAECs and HSC amplification and indicate the potential role of arterial vascular niche to decode the in vivo information for self-renewal and expansion of human HSCs.

## Materials and methods

### Isolation and culture of umbilical cord arterial/vein endothelial cells

The umbilical cord was collected by the Beijing Yuhe Chinese and Western Medicine Integrative Rehabilitation Hospital (ZXYEC-KT-2017-04-P01). Primary HuAECs and human umbilical vein endothelial cells (HuVECs) were isolated as previously described [21, 22]. A sterile technique was utilized in all manipulations of the cord. The cord was separated from the placenta soon after birth and stored in a sterile container filled with DMEM (Gibco, Big island, NY, USA) at 4 °C until processing. Storage time averaged about 2 h, and cords were discarded if held more than 6 h. Briefly, the umbilical cord arteries and vein were dissociated out and rapidly placed in preheated phosphate buffer saline (PBS; Gibco, Big island, NY, USA), and a 20-cm clipping of the tissue was used for cell isolation. The arteries and vein were perfused with PBS to wash out the blood and allowed to drain. The one end of the umbilical arteries/vein was then cannulated with a syringe clamped shut with a hemostat. Then the arteries and vein were infused with Collagenase IV (1 mg/ml; Sigma-Aldrich, Shanghai, China) for 15 min of incubation at 37 °C after the other end of blood vessel was secured with a hemostat. After incubation, the collagenase solution containing the ECs was flushed from the cord by perfusion with PBS in a sterile 50-ml conical centrifuge tube and centrifuged at 1000 rpm for 5 min. Subsequently, the cell pellets were resuspended in EGM-2 medium (Lonza, Beijing, China) and incubated at 37 °C under 5% C0_2_. The cells were fed twice a week with a complete change of fresh culture medium. The umbilical cord samples used to isolate PECs were from several donors. The primary HuAECs and HuVECs from the same donor were paired for independent experiments.

### Virus preparation and transfection

HuAECs-E4orf1-GFP and engineered human umbilical vein endothelial cells [20] (HuVECs-E4orf1-GFP) were generated by introducing a retroviral vector into primary HuAECs and HuVECs. Retrovirus was generated by transfecting MSCV-N *E4ORF1* (Addgene, Shanghai, China; species, human adenovirus 5; size, 384 bp plus 8162 bp; vector type, mammalian expression, retroviral; selectable markers, puromycin) and pMX-*GFP* (provided by Dr. Hiroyuki Hirai, USA) in Plat A cells using Lipofectamine 2000 (Invitrogen, Carlsbad, California, USA). Retroviral constructs were collected 44 and 68 h post-transfection. *E4ORF1*-transfected ECs were selected with 0.5 μg/ml puromycin (InvivoGen, Shanghai, China). The *DLL4* shRNA and control shRNA (both carry GFP label) were designed by Genechem (Shanghai, China) and transfected individually into primary HuAECs. Transfected GFP^+^ cells were enriched via fluorescence-activated cell sorting (FACS) Verse flow cytometer (BD Biosciences, Franklin, NJ, USA). Virus transfection experiments were performed on PECs from three different donors.

### Flow cytometry (FCM)

Flow cytometric analysis was performed using the following antibodies: CD144-PE, CD45-APC, CD133-PE, CD31-APC, and CD309-PE for primary HuAECs and HuVECs; FVS510, CD34-PE, CD38-APC, and CD90-PE-cy7 for ex vivo cultured assays; and anti-human CD45-APC, CD19-APC, CD11b-PerCP-CY5.5, and anti-mouse CD45.1-FITC for in vivo transplantation experiments. Cells were stained at 4 °C for 40 min, light protected. The filtered (70 μm) samples were analyzed on the FACSVerse flow cytometer. All antibodies are from BD Biosciences (Franklin, NJ, USA) or eBioscience (San Diego, CA, USA).

### Immunofluorescence

Primary HuAECs and HuVECs were stained for confirmation of cell identity. The cultures were fixed in 4% paraformaldehyde (Sigma-Aldrich, Shanghai, China), permeabilized and blocked, and then incubated overnight in blocking solution containing primary antibody against von Willebrand Factor (vWF; 1:500; Sino Biological, Beijing, China). FITC-conjugated Goat anti-rabbit IgG (1:200; Beijing Zhongshan Jinqiao Biological Technology, Beijing, China) was used as a secondary antibody and DAPI (1 mg/mL; Roche, Basel, Switzerland) as a nuclei counterstain. Imaging was performed using confocal microscopy (PerkinElmer, Waltham, MA, USA) and Volocity Software (PerkinElmer, Waltham, MA, USA).

### Tube formation assay

Based on previously described [23], the primary HuAECs and HuVECs suspended in EGM-2 medium supplemented with VEGF (100 ng/ml; R&D Systems, Aimolivel, California, CA, USA) were seeded into 6-well plates coated with Matrigel (BD Biosciences, Franklin, NJ, USA) prior at a density of 10,000 cells/cm^2^. After 24 h of incubation, cells were photographed using confocal microscopy and Volocity Software.

### Quantitative real-time polymerase chain reaction (qRT-PCR) analysis

Total RNA was extracted from cells by using RNeasy Micro Kit (QIAGEN, New York, NY, USA) and reverse transcribed by using ReverTra Ace qPCR RT Master Mix (TOYOBO, Shanghai, China) according to the manufacturer’s specifications. The PCR products were detected using THUNDERBIRD SYBR qPCR Mix (TOYOBO, Shanghai, China). The primer sequences used in qRT-PCR assays are shown in Table 1.

**Table 1:** Quantitative RT-PCR primer sequences

Gene	Sequence (5′-3′)
*CXCR4*	F:GCCTTATCCTGCCTGGTATTGTC
	R:GCGAAGAAAGCCAGGATGAGGAT
*DLL1*	F:GACGAACACTACTACGGAGAGG
	R:AGCCAGGGTTGCACACTTT
*DLL4*	F:TGGGTCAGAACTGGTTATTGGA
	R:GTCATTGCGCTTCTTGCACAG
*EFNB2*	F:TTCAGCCCTAACCTCTGGGG
	R:CCTCCAAAGACCCATTTGATGTA
*GJA4*	F:TGCAAGAGTGTGCTAGAGGC
	R:ACAAAGCAGTCCACGAGGTAG
*HEY1*	F:GTTCGGCTCTAGGTTCCATGT
	R:CGTCGGCGCTTCTCAATTATTC
*HEY2*	F:CCTAACAGAAGTTGCGCGGTA
	R:GAGGCGACAAGGGGTTGAC
*JAG1*	F:GTCCATGCAGAACGTGAACG
	R:GCGGGACTGATACTCCTTGA
*NOTCH1*	F:GAGGCGTGGCAGACTATGC
	R:CTTGTACTCCGTCAGCGTGA
*NOTCH2*	F:CAACCGCAATGGAGGCTATG
	R:GCGAAGGCACAATCATCAATGTT
*NOTCH3*	F:CGTGGCTACACTGGACCTC
	R:AGATACAGGTGAACTGGCCTAT
*NOTCH4*	F:TGTGAACGTGATGTCAACGAG
	R:ACAGTCTGGGCCTATGAAACC
*APLNR*	F:CCTGCATCAGCTACGTCAACA
	R:GGGATGGATTTCTCGTGCATCT
*EPHB4*	F:CGCACCTACGAAGTGTGTGA
	R:GTCCGCATCGCTCTCATAGTA
*NR2F2*	F:AACCAGCCGACGAGATTCG
	R:CCCGGATGAGGGTTTCGATG
*NRP2*	F:CCAACGGGACCATCGAATCTC
	R:CCAGCCAATCGTACTTGCAGT
*E4ORF1*	F:CGCCGGAATTAGATCTGCCA
	R:CTCGAGCAGCGTAATCTGGA
*ANGPTL4*	F:GGCTCAGTGGACTTCAACCG
	R:CCGTGATGCTATGCACCTTCT
*IGF2*	F:GTGGCATCGTTGAGGAGTG
	R:CACGTCCCTCTCGGACTTG
*HOXB4*	F:GCACGGTAAACCCCAATTA
	R:GGCAACTTGTGGTCTTTTTT
*GATA2*	F:GCAACCCCTACTATGCCAACC
	R:CAGTGGCGTCTTGGAGAAG
*RUNX1*	F:ATGTGGTCCTATTTAAGCCAGCCC
	R:TCATCTGGCTGAAGACACCAGCTT
*HES1*	F:TCAACACGACACCGGATAAAC
	R:GCCGCGAGCTATCTTTCTTCA
*GAPDH*	F:GAGTCAACGGATTTGGTCGT
	R:TTGATTTTGGAGGGATCTCG

### Nitric oxide production assay

Based on the previous description [24], primary HuAECs and HuVECs were seeded into vitronectin-precoated 24-well plates (100,000 cells/well). After 2 days, the cultured media were changed to fresh EGM-2 containing DAF-FM (1 μM; Life Technologies, Carlsbad, CA, USA). The cells were cultured for 30 min at 37 °C and then harvested for flow cytometric analysis.

### Ex vivo coculture

The sample of human CB was collected by the Beijing Yuhe Chinese and Western Medicine Integrative Rehabilitation Hospital (ZXYEC-KT-2017-04-P01). After density gradient centrifugation and immunomagnetic selection (Miltenyi Biotec, Westphalia, Gladbach, Germany), a total of 50,000 CD34^+^ cells were cultured in StemSpan (STEMCELL Technologies, Shanghai, China) containing 50 ng/mL of rhSCF, rhTPO, and rhFlt-3 L (PeproTech, Rocky Hill, NJ, USA), with HuAECs-E4orf1-GFP and HuVECs-E4orf1-GFP or without feeder cells (cytokines alone). Additionally, we also performed a Notch signaling blocking experiment in hCB cells cocultured with HuAECs-E4orf1-GFP by adding Compound E (200 nM; CpE; MERCK, Darmstadt, Germany), which is a Notch signal inhibitor. CpE was added every other day and an equivalent dose of DMSO was added as a control. In order to investigate the effect of *DLL4* in the coculture system, the CD34^+^ hCB cells were cocultured with HuAECs-E4orf1-shDLL4 and its control group (HuAECs-E4orf1 carry unrecognized sequence and GFP). After 14 days, the expanded hCB cells were harvested for analyses.

### Colony-forming unit (CFU) assay

For colony-forming assays, 250 amplified hCB cells were seeded into 24-well plates containing MethoCult H4434 (STEMCELL Technologies, Shanghai, China). Each group was performed in triplicate. The colonies including CFU-erythrocyte (CFU-E), burst-forming unit-erythroid (BFU-E), CFU-granulocyte (CFU-G), CFU-granulocyte, macrophage (CFU-GM), and CFU-megakaryocyte (CFU-M) emerged from day 7 and were scored on day 12.

### Giemsa staining

Colony-forming cells (CFCs) were assessed by means of the Giemsa staining kit (Baso, Zhuhai, China), according to the manufacturer’s protocols. Equivolumetric cells were collected and distributed onto slides. Then, solution A was added and allowed to act for 1 min at room temperature. After that, solution B was added and incubated for further 10 min. Washed and air dried the samples before recording.

### In vivo transplantation

All mice experiments were approved by the Institutional Animal Care and Use Committee (IACUC) at Institute of Health Service and Transfusion Medicine (Reference number: IACUC of AMMS-13-2016-016). Briefly, 6-week-old NSG mice were sublethally total body irradiated (2.5 Gy) and injected 1,000,000 expanded hCB cells from each group through the tail vein intravenously. At 16 weeks, the peripheral blood (PB) cells/femurs/tibias/spleens were collected and the percentage of human CD45^+^ cells was assessed.

### Statistical analysis

Results are expressed as mean ± standard deviation. *P* value less than 0.05 (two-tailed Student’s *t* test) was considered statistically significant. The “n” stands for biological replicates and all those repeats were independent.

## Results

### Molecular and functional characterization of HuAECs

**Fig. 1:**
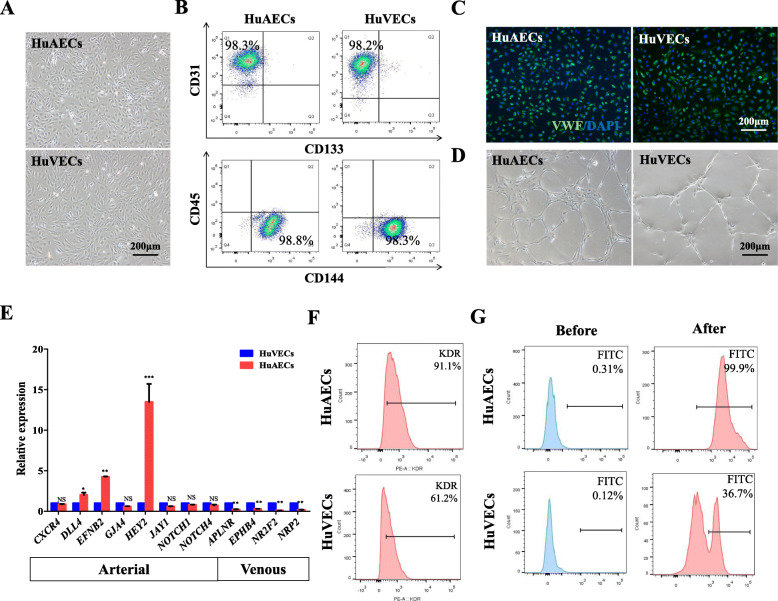
Isolation and identification of arterial and vein endothelial cells. **a** Primary HuAECs and HuVECs were isolated and cultured. **b** Flow cytometric analysis of CD31, CD133, CD144, and CD45 expression in primary endothelial cells. **c** Fluorescent images confirmed that HuAECs and HuVECs expressed vWF. **d** HuAECs and HuVECs formed a tube cavity structure in vitro. **e** qRT-PCR analysis of arterial and venous markers expression in HuAECs and HuVECs. **f** Flow cytometric analysis showed that KDR expression in HuAECs is higher than in HuVECs. **g** NO production was evaluated by the intensity of DAF-FM which form fluorescent wavelengths similar to FITC. NS, no significant difference, *P < 0.05, **P < 0.01, ***P < 0.001; *n* = 3; scale bar, 200 μm

Flow cytometric analysis was used to characterize cell surface markers on the subcultured HuAECs (Fig. 1a), showing that the HuAECs were positive for endothelial cell markers, such as CD31 and CD144, but negative for CD133 and CD45 (Fig. 1b), which suggested that the cells were free of endothelial progenitor cells (CD133^+^ cell population) or mature blood cells (CD45^+^ cell population). HuVECs were also tested as controls. The expression of vWF, a common marker for human endothelium, was identified by immunofluorescence (Fig. 1c, Supplement Fig. 1a). In addition, tube formation assay was tested and the results showed that HuAECs plated in Matrigel can form capillary-like structures (Fig. 1d). qRT-PCR revealed that, compared with HuVECs, HuAECs expressed several arterial genes, including *DLL4*, *EFNB2*, and *HEY2* [25], whereas the venous master regulators were greatly diminished, such as *APLNR*, *EPHB4*, *NR2F2*, and *NRP2* [23] (Fig. 1e). To better understand their expression profile, we further analyzed the expression of another key AEC marker, KDR [26]. We found that HuAECs highly express KDR compared with HuVECs (Fig. 1f). We also performed analyses of arterial-specific functions, such as higher NO oxide production [27, 28], by comparing HuAECs with HuVECs. NO production was revealed by the intensity of DAF-FM. DAF-FM is nonfluorescent until it reacts with NO to form a fluorescent benzotriazole. The fluorescent intensity was measured by FCM and the results demonstrated that HuAECs produced higher NO than HuVECs (Fig. 1g).

### Establishment and identification of HuAECs-E4orf1-GFP feeders

**Fig. 2:**
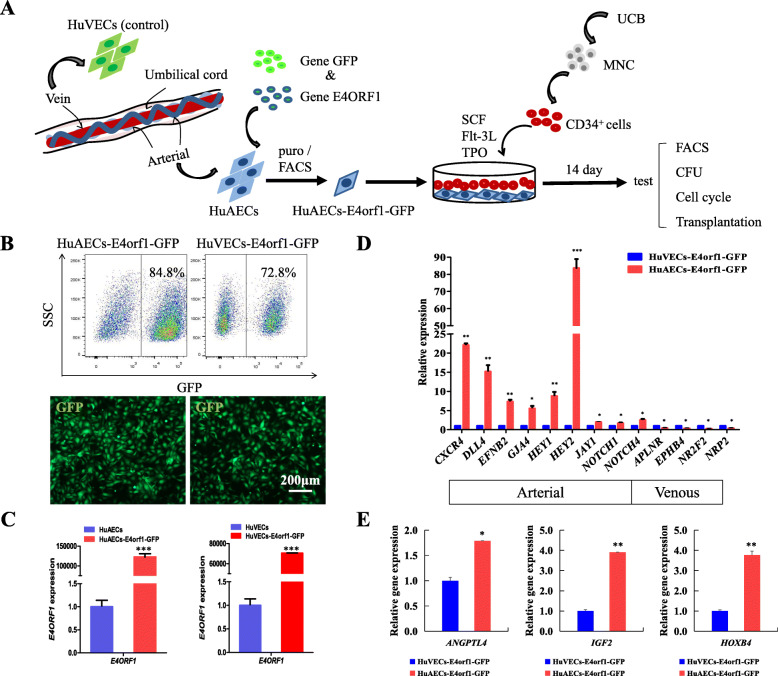
Generation of engineered human umbilical arterial endothelial cells by transduction of adenoviral *E4orf1* and *GFP* (HuAECs-E4orf1-GFP). **a** Schematic illustration of the protocol for test our hypothesis on the role of HuAECs in supporting ex vivo HSPC expansion. **b** Fluorescence intensity of GFP in HuAECs-E4orf1-GFP and HuVECs-E4orf1-GFP before FACS (above panel); the image of GFP expression in HuAECs-E4orf1-GFP and HuVECs-E4orf1-GFP after FACS (button panel). **c** Detection of *E4ORF1* expression in HuAECs-E4orf1-GFP and HuVECs-E4orf1-GFP by qRT-PCR. **d** qRT-PCR analysis of arterial and venous markers expression in HuAECs-E4orf1-GFP and HuVECs-E4orf1-GFP. **e** Comparison of the relative expression of *ANGPTL4*, *IGF2*, and *HOXB4* in HuAECs-E4orf1-GFP and HuVECs-E4orf1-GFP. *P < 0.05, **P < 0.01 ***P < 0.001; *n* = 3, scale bar, 200 μm

To test our hypothesis on the role of HuAECs in supporting ex vivo HSPC expansion, we isolated hCB CD34^+^ cells and cultured it in rhSCF, rhFlt3L, and rhTPO with or without feeder cells. The whole strategy is illustrated in Fig. 2a. The HuAECs-E4orf1-GFP were constructed by transduction of the HuAECs with the recombined retroviral vector MSCV-N *E4ORF1* and pMX-*GFP*. Then, the transgenic cell strains were further purified with 0.5 μg/ml puromycin selection for 3–5 days and FACS by *GFP* expression (Fig. 2b), with primary HuAECs as a control. HuAECs-E4orf1-GFP still maintain stable cell phenotype within 10 generations (Supplement Fig. 1B, C and D). To determine the expression of *E4ORF1* in HuAECs-E4orf1-GFP, qRT-PCR was used to detect the expression level of *E4orf1* mRNA in HuAECs-E4orf1-GFP or HuAECs. As shown in Fig. 2c, *E4ORF1* was highly expressed in HuAECs-E4orf1-GFP feeders but barely in primary HuAECs. Briefly, HuAECs-E4orf1-GFP maintain the expression of arterial genes in serum/cytokine-free culture conditions (Fig. 2d). In contrast, primary HuAECs rapidly undergo apoptosis in serum/cytokine-free culture conditions (Supplementary Fig. 3A). On the other hand, primary HuAECs gradually decreased the AEC-specific gene expression with the increase of generations in the utilization of enriched EC growth medium supplemented with serum (Supplementary Fig. 3B). Besides, HuAECs-E4orf1-GFP expressed a higher level of growth factors and signaling molecules that support HSC expansion, including *ANGPTL4* [29], *IGF2* [30], and *HOXB4* [31], compared with HuVECs-E4orf1-GFP (Fig. 2e).

### Coculture of HuAECs-E4orf1-GFP enhanced ex vivo expansion of CB HSPCs while heightened the multilineage differentiation potential of HSPCs in vitro

**Fig. 3:**
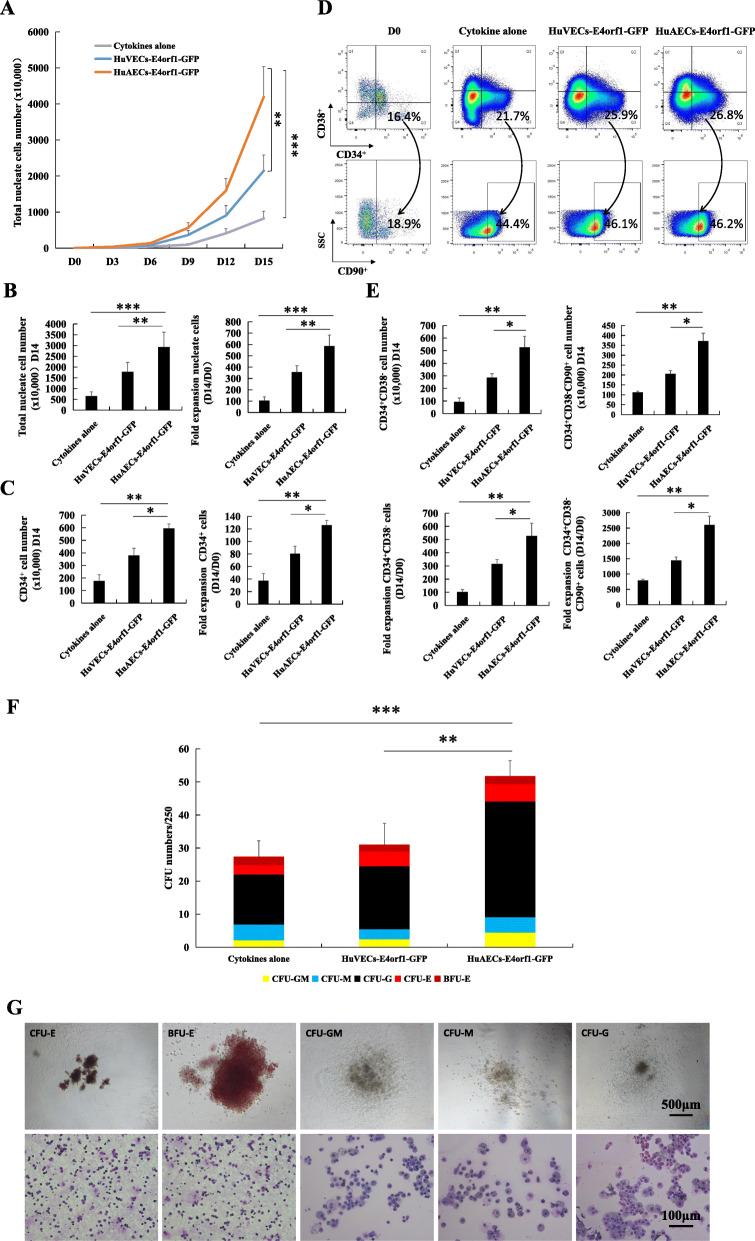
CD34^+^ hCB cells cocultured with HuAECs-E4orf1-GFP resulted in a significant expansion of HSPCs while heightened in vitro multilineage differentiation potential. **a** The cumulative curve of TNCs during the expansion. **b** TNC expansion. **c** Total CD34^+^ hematopoietic cell expansion. **d** Representative scatter plots of CD34^+^CD38^−^CD90^+^ cell expansion. **e** The number of CD34^+^CD38^−^ cells (left panel) and CD34^+^CD38^−^CD90^+^ cells (right panel) significantly increased in the HuAECs-E4orf1-GFP group at day 14. **f** CFU numbers of amplified CD34^+^ hCB cells in different groups after 14-day expansion. **g** CFU (CFU-E, BFU-E, CFU-GM, CFU-M, and CFU-G) morphology (above panel, scale bar: 500 μm) and Giemsa’s staining for cytospin samples of CFUs (bottom panel, scale bar: 100 μm). *P < 0.05, **P < 0.01, ***P < 0.001; *n* = 8. Three HuAECs-E4orf1-GFP and HuVECs-E4orf1-GFP cell lines were established and all of them have stable and consistent effects on ex vivo expansion of HSPCs

To test the effect of HuAECs-E4orf1-GFP on the ex vivo expansion of HSPCs, the CD34^+^ hCB cells were cumulatively expanded with HuAECs-E4orf1-GFP, HuVECs-E4orf1-GFP, or without feeders under serum-free and minimal cytokine conditions that incorporated rhSCF, rhTPO, and rhFlt3 as growth factors. The feeder cells used for coculture system were all within 6–10 passages. As a result, the CD34^+^ hCB cells cocultured with HuAECs-E4orf1-GFP resulted in significant augmentation of total nucleated cells (TNCs) and CD34^+^ cells than cytokines alone culture or HuVECs-E4orf1-GFP coculture (Fig. 3a–c). Moreover, the number of CD34^+^CD38^−^ cells and more primitive CD34^+^CD38^−^CD90^+^ cells in expanded cells cocultured with HuAECs-E4orf1-GFP increased 527.2 ± 96.3 and 2603.4 ± 283.2-folds within 2 weeks, which was 6.6 ± 2.5 and 3.3 ± 0.8 times of the cytokines alone group, 1.9 ± 0.5 and 1.8 ± 0.3 times of the HuVECs-E4orf1-GFP group (Fig. 3d, e). To assess the in vitro capability of multilineage differentiation in expanded CD34^+^ hCB cells, CFU assay was performed. Compared with the other two groups, HuAECs-E4orf1-GFP coculture significantly increased numbers of CFU, demonstrating that the colony-forming potential of HSPCs was heightened in vitro in this condition (Fig. 3f, g).

### Expanded cells maintained repopulating activity in NSG mice in vivo

**Fig. 4:**
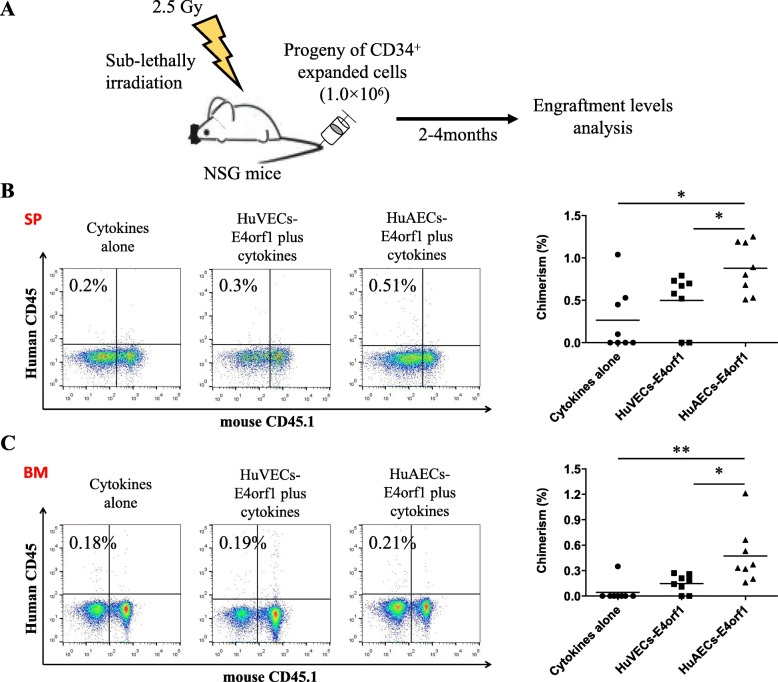
CD34^+^ hCB cells cocultured with HuAECs-E4orf1-GFP have repopulating capability in vivo. **a** Schematic illustration of the protocol for in vivo transplantation. NSG mice were transplanted with 1,000,000 cells from each culture after sublethally irradiation. **b** Representative scatter plots of human CD45^+^ hematopoietic cell engraftment in mouse’s spleen after 16-week transplantation (left panel). Chimerism of engrafted human CD45^+^ hematopoietic cells in mouse’s spleen after 16-week transplantation (right panel). **c** Representative scatter plots of human CD45^+^ hematopoietic cell engraftment in mouse’s BM after 16-week transplantation (left panel). Chimerism of engrafted human CD45^+^ hematopoietic cells in mouse’s BM after 16-week transplantation (right panel). Formula: chimerism (%) = [%hCD45^+^/(%hCD45^+^+%mCD45^+^)]. *P < 0.05, **P < 0.01; *n* = 8

In order to examine the hematopoietic reconstitution capacity in vivo, 1,000,000 progeny of ex vivo expanded CD34^+^ hCB cells from HuAECs-E4orf1-GFP coculture, HuVECs-E4orf1-GFP coculture or cytokine alone culture were transplanted into sublethally irradiated (2.5 Gy) NSG mice (Fig. 4a). Control mice were injected with PBS. Chimerism ratios were determined by flow cytometric analysis as the percentage of human CD45^+^ cells in PB, BM, and the spleen of NSG mice. Mice with more than 0.1% human cells were considered positive. We observed that the HuAECs-E4orf1-GFP-expanded group resulted in higher engraftment of human CD45^+^ cells than the HuVECs-E4orf1-GFP coculture or cytokine alone culture in PB after 4 weeks (frequency of engraftment, 4/8), 8 weeks (frequency of engraftment, 8/8), or 16 weeks (frequency of engraftment, 8/8) transplantation (Supplementary Fig. 4B). Furthermore, cocultured CD34^+^ hCB cells with HuAECs-E4orf1-GFP more efficiently engrafted human CD45^+^ cells at 16 weeks in mouse’s spleen and BM after transplantation than that in CD34^+^ hCB cells expanded with HuVECs-E4orf1-GFP or cytokines alone (Fig. 4b, c). These results show that HuAECs-E4orf1-GFP support the expansion of CD34^+^ hCB cells which are capable of maintaining the long-term repopulating ability of NSG mice, while multilineage hematopoietic engraftment was also observed after 16-week transplantation (Supplementary Fig. 4A).

### Role of Notch signaling pathway in HSPC expansion

**Fig. 5:**
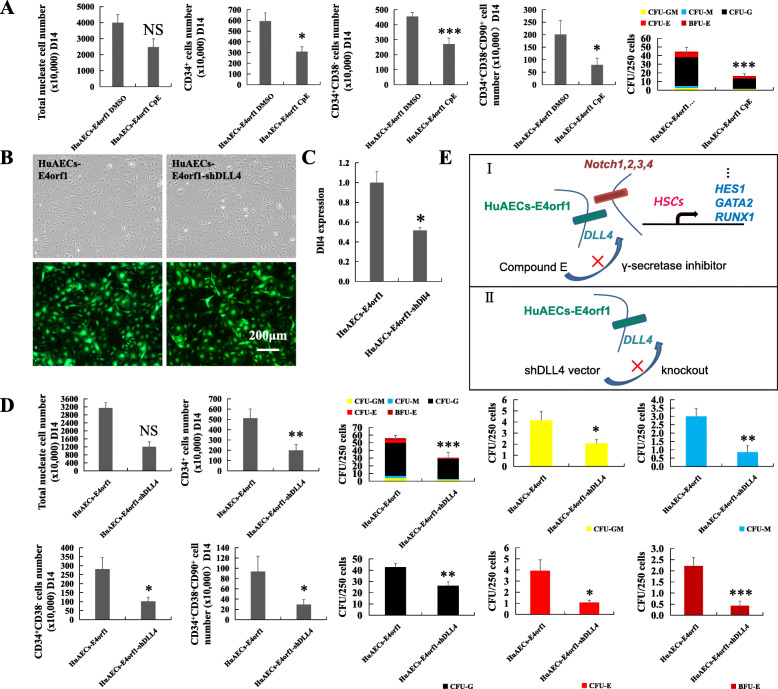
CD34^+^ hCB cells amplified on HuAECs-E4orf1-GFP exhibited Notch signaling activation. **a** The CD34^+^ hCB cell expansion at day 14 and CFU formation in CpE treatment group or with a vehicle control (DMSO) group. **b** Image of GFP expression in HuAECs-E4orf1-shLV6 (left panel) and HuAECs-E4orf1-shDLL4 (right panel) after FACS. **c** Expression of *DLL4* in HuAECs-E4orf1-shLV6 and HuAECs-E4orf1-shDLL4. **d** The number of hematopoietic cells and CFU decreased in the HuAECs-E4orf1-shDLL4 group. **e** Hypothesis for the signal pathways involved in DLL4-mediated promoting proliferation activity of HuAECs-E4orf1 was indicated. NS, no significant difference, *P < 0.05, **P < 0.01, ***P < 0.001; *n* = 8; scale bar, 200 μm

DLL4 plays a critical role in activating the Notch pathway associated with proliferation and maturation of HSCs [32]. Interestingly, HuAECs-E4orf1-GFP express high levels of *DLL4* compared to HuVECs-E4orf1-GFP. In view of this, we speculated that HuAECs-E4orf1-GFP might support ex vivo HSPC expansion through Notch signaling activation. For further verification, a specific Notch signaling inhibitor (CpE) was used in our expansion system and the results showed that the growth-promoting activity of HuAECs-E4orf1-GFP on the expanded CD34^+^ hCB cells was obviously hampered, reflected in TNCs, CD34^+^, CD34^+^CD38^−^, CD34^+^CD38^−^CD90^+^ cell, and even CFU numbers (Fig. 5a). Next, we further analyzed whether the reduced expression of *DLL4* in HuAECs-E4orf1-GFP would affect HSPC expansion in coculture system. Simultaneously, an *DLL4* knockdown coculture system was utilized to investigate the precise effects of *DLL4* in HuAECs-E4orf1-GFP. HuAECs-E4orf1 were stably transfected with shDLL4 and negative control shRNA (Fig. 5b). qRT-PCR confirmed that *DLL4* expression was effectively inhibited in HuAECs-E4orf1-shDLL4 (Fig. 5c). In this experiment, the suppression of cell proliferation and CFU formation was significantly greater in HuAECs-E4orf1-shDLL4 coculture (Fig. 5d). Consistently, the CD34^+^ hCB cells amplified on HuAECs-E4orf1-GFP exhibited a higher level of Notch ligands, receptors, and target gene (*GATA2*, *HES1*, and *RUNX1*) transcription than cells with cytokines alone or HuVECs-E4orf1-GFP coculture on the 14th day of amplification (Supplementary Fig. 3C, D).

## Discussion

Despite decades of efforts to explore the strategy of ex vivo HSC expansion, defining a set of cytokines that can expand long-term engraftable human HSCs has proven to be difficult so far [33]. The crux of this problem may lie in our ultimate incomprehension on the manipulation of HSCs by in vivo niche. Here we hypothesized HuAECs could act as a vascular niche to ex vivo expand HSPCs by providing prohematopoietic signals. To test this hypothesis, we established human umbilical cord arterial endothelial feeders by introducing the adenoviral *E4ORF1* and *GFP* gene using a retrovirus vector. We took the lead in suggesting that HuAECs-E4orf1-GFP provided a suitable cellular environment for effective expansion of CD34^+^ hCB cells with multipotency in vitro and engraftment capability in vivo.

In the present study, isolated HuAECs sustained in our cultures exhibited an AEC-surface expressional profile. Despite the well-known role for *E4ORF1* in supporting long-term survival of primary endothelial cells in the absence of serum and angiogenic factors [34], this is the first report that *E4ORF1* is directly involved in backing up the in vitro arterial identity. These studies make a case for retaining arterial genes and signaling, involving ligands DLL4 [35], receptors NOTCH1/4 [36], and transcription factors (TFs) HEY1/2 [25, 37] as a major determinant of AEC identity.

A requirement for complex signaling networks in ex vivo HSC expansion has motivated the development of cellular-based platforms. According to our current knowledge, mesenchymal stromal cells (MSCs) [38], osteoblasts [39], and other stromal cells, including sinusoidal cells and endothelial cells [40, 41], are responsible for supporting hematopoiesis and controlling HSC numbers. Consequently, mesenchymal progenitor cells [42, 43] and endothelial cells [44, 45] have been widely used as surrogates for mesenchymal niche and endothelial niche to the expansion of HSPCs. Previously, we comprehensively characterized liver sinusoidal endothelial cells from the human fetal liver (hFLSECs) and found that they enable effective expansion of CD34^+^ hCB cells with multipotency in vitro and engraftment capability in NSG mice [46]. As we all know, the fetal liver serves as a predominant site for expansion of functional HSCs during embryogenesis [47], which makes much more sense about the important role of hFLSECs on the expansion of CD34^+^ hCB cells. In this study, we compared the capacity of hFLSECs and HuAECs to facilitate the expansion of CD34^+^ hCB cells. Encouragingly, HuAECs has the advantages over hFLSECs in growth-promoting activity and capability of CFU formation (Supplementary Fig. 2).

ECs lining arteries and veins have distinct molecular signatures and functions. Ligands *DLL1/4*, receptors *NOTCH1/4*, and TFs *HEY1/2* are major determinants of arterial EC identity [48–51]. As is well-known, hemogenic endothelium (HE) is associated with arteries. Former studies suggested that DLL4^low/−^ cells within HE receive Notch-activating signals from DLL4^high^ cells, sparking off an endothelial-to-hematopoietic transition [52]. Additionally, definitive hematopoiesis allied to endothelial cell development and arterial specification is regulated by the conserved signaling pathways Sonic Hedgehog, Wnt, and Notch, with a prominent role for the ligand DLL4 [35, 36, 53, 54]. In our study, we identified HuAECs-E4orf1-GFP expressed high levels of arterial genes, including *DLL4*, *EFNB2*, and *HEY2* in comparison with HuVECs-E4orf1-GFP. Notably, the supplement of CpE, a specific Notch signaling inhibitor, significantly hampered the growth-promoting activity of HuAECs-E4orf1-GFP. In view of the well-known role for *DLL4* in arterial development, in vitro loss of function analyses were subsequently performed in HuAECs-E4orf1-GFP. The results showed that knockdown of *DLL4* remarkably decreased the proliferation and CFU formation of HSPCs in coculture system, implicating that *DLL4* may be one of the candidate elements for supporting HSC expansion (Fig. 5e). These results may lay a foundation to further uncover the mechanisms of cross-talk between ECs and HSC during amplification.

Coculture of HSCs with stromal cells and growth factors has been utilized in an attempt to ex vivo recapitulate interactions in hematopoietic microenvironment to expand HSPCs. For instance, mesenchymal stromal cell (MSC)-mediated expansion is one of the current approaches to the ex vivo manipulation of CB stem cells in clinical trials. Here we developed a new human CB expansion platform, and our study further confirmed the potential role for cell-based HSPC expansion methodologies. In our report, the primary HuAECs and HuVECs from the same donor were paired for independent experiments while the umbilical cord samples used to isolate PECs were from several donors. The different sources contained herein indicated that this coculture system is of universal applicability. In future, infusion of off-the-shelf HuAEC-mediated ex vivo expanded CB-derived HPSCs might be developed a novel strategy for dealing with the shortage of HSPCs. On the other hand, this platform might also allow identification of new growth factors or targeted defined molecules, contributing to define a set of cytokines for application in HSC expansion.

## Conclusion

In summary, we identified that HuAECs acted as a potential cellular platform to efficiently support the expansion of engraftable human CB HSCs. Despite the well-known role for endothelium in the initial generation of HSCs [55], HSC augment [56], and hematopoietic lineage-specific differentiation [52], this is the first time that arterial endothelial cells are directly involved in the ex vivo expansion of human HSCs. Ultimately, a better understanding of the cellular and signaling components in this region will advance the expansion of HSCs for therapeutic purposes.

## Supplementary information

**Additional file 1: Supplementary Fig. 1.** The endothelial cell phenotype retained within 10 passages after transfection. (A) vWF staining negative control. (B) Flow cytometric analysis of CD31 and KDR expression in HuVECs-E4orf1-GFP. (C) Flow cytometric analysis of CD31 and KDR expression in HuAECs-E4orf1-GFP. (D) qRT-PCR analysis of arterial and venous markers expression in HuVECs-E4orf1-GFP and HuAECs-E4orf1-GFP for different generations. *P < 0.05, **P < 0.01, ***P < 0.001; *n* = 3; Scale bar: 200 μm.

**Additional file 2: Supplementary Fig. 2.** Contrasting the effect of HuAECs-E4orf1-GFP and hFLSECs-E4orf1-GFP on CD34^+^ hCB cell expansion. (A) TNC expansion at day 14. (B) The cumulative curve of TNC in ex vivo expansion. (C) CD34^+^CD38^−^ (left panel) and CD34^+^CD90^+^ cell (right panel) expansion. (D) CFU number of amplified CD34^+^ hCB cells in HuAECs-E4orf1-GFP coculture or hFLSECs-E4orf1-GFP coculture. NS means ‘no significant difference’, *P < 0.05, **P < 0.01; *n* = 5.

**Additional file 3: Supplementary Fig. 3.** Gene expression of PECs and expanded CD34^+^ hCB cells. (A) The morphology of PECs cultured in serum-free, cytokine-free condition. Scale bar: 200 μm. (B) qRT-PCR analysis of arterial and venous markers expression in primary HuVECs and HuAECs for different generations. (C) Comparison of the relative transcript levels of Notch ligands and receptors in expanded CD34^+^ hCB cells from different groups at day 14. (D) The transcript levels of Notch target genes (*GATA2, HES1* and *RUNX1*) in expanded CD34^+^ hCB cells at day 14. NS means ‘no significant difference’, *P < 0.05, **P < 0.01, ***P < 0.001; *n* = 3.

**Additional file 4: Supplementary Fig. 4.** Multilineage engraftment measurements following transplantation. (A) Multilineage human hematopoietic cell engraftment after 16-week transplantation. (B) Percentage of engrafted human CD45^+^ hematopoietic cells at 4, 8 and 16 weeks after transplantation. *n* = 8.

## Data Availability

Not applicable

## Abbreviations

CB: Cord blood
HSC: Hematopoietic stem cell
HSPCs: Hematopoietic stem and progenitor cells
HuAECs-E4orf1-GFP: Engineered human umbilical arterial endothelial cell
HuVECs: Human umbilical vein endothelial cells
HSCs: Hematopoietic stem cells
PSC: Pluripotent stem cells
iPSC: Induced pluripotent stem cells
ESC: Embryonic stem cells
BM: Bone marrow
UCB: Umbilical cord blood
MPB: Mobilized peripheral blood
ECs: Endothelial cells
AGM: Aorta-gonad-mesonephros
AECs: Arterial endothelial cells
HuAEC: Human umbilical arterial endothelial cell
GFP: Green fluorescent protein
PECs: Primary endothelial cells
NSG: NOD.Cg-Prkdc^scid^Il2rg^tm1Wjl^/SzJ
PBS: Phosphate buffer saline
HuVECs-E4orf1-GFP: Engineered human umbilical vein endothelial cells
FACS: Fluorescence-activated cell sorting
vWF: von Willebrand Factor
qRT-PCR: Quantitative real-time polymerase chain reaction
CpE: Compound E
CFU: Colony-forming unit
CFU-E: CFU-erythrocyte
BFU-E: Burst-forming unit-erythroid
CFU-G: CFU-granulocyte
CFU-GM: CFU-granulocyte, macrophage
CFU-M: CFU-megakaryocyte
CFCs: Colony-forming cells
IACUC: Institutional Animal Care and Use Committee
PB: Peripheral blood
TNCs: Total nucleated cells
hFLSECs: Human fetal liver
HE: Hemogenic endothelium
