# Supplementary figures and images for: Arterial endothelium creates a permissive niche for expansion of human cord blood hematopoietic stem and progenitor cells
Source: Stem Cell Res Ther. 2020 Aug 14;11:358. doi: 10.1186/s13287-020-01880-8 (PMC7429738; doi:10.1186/s13287-020-01880-8)

# Supplementary Figure 1

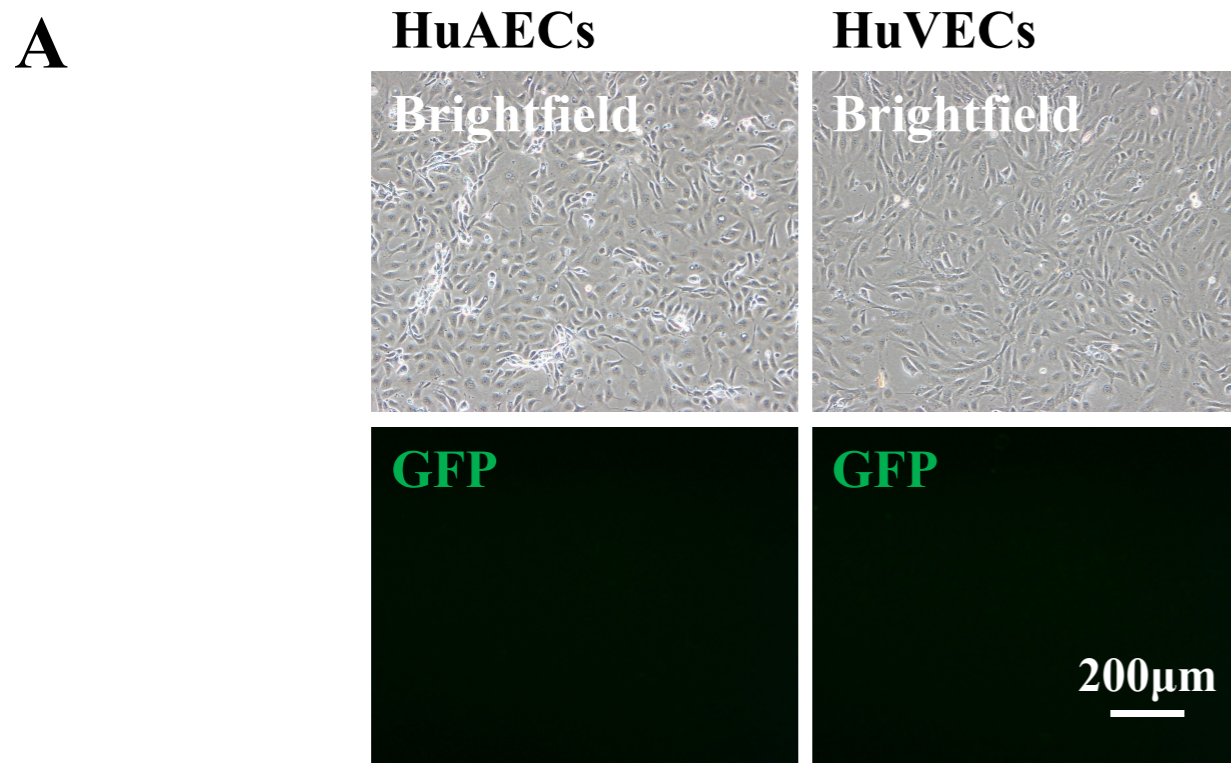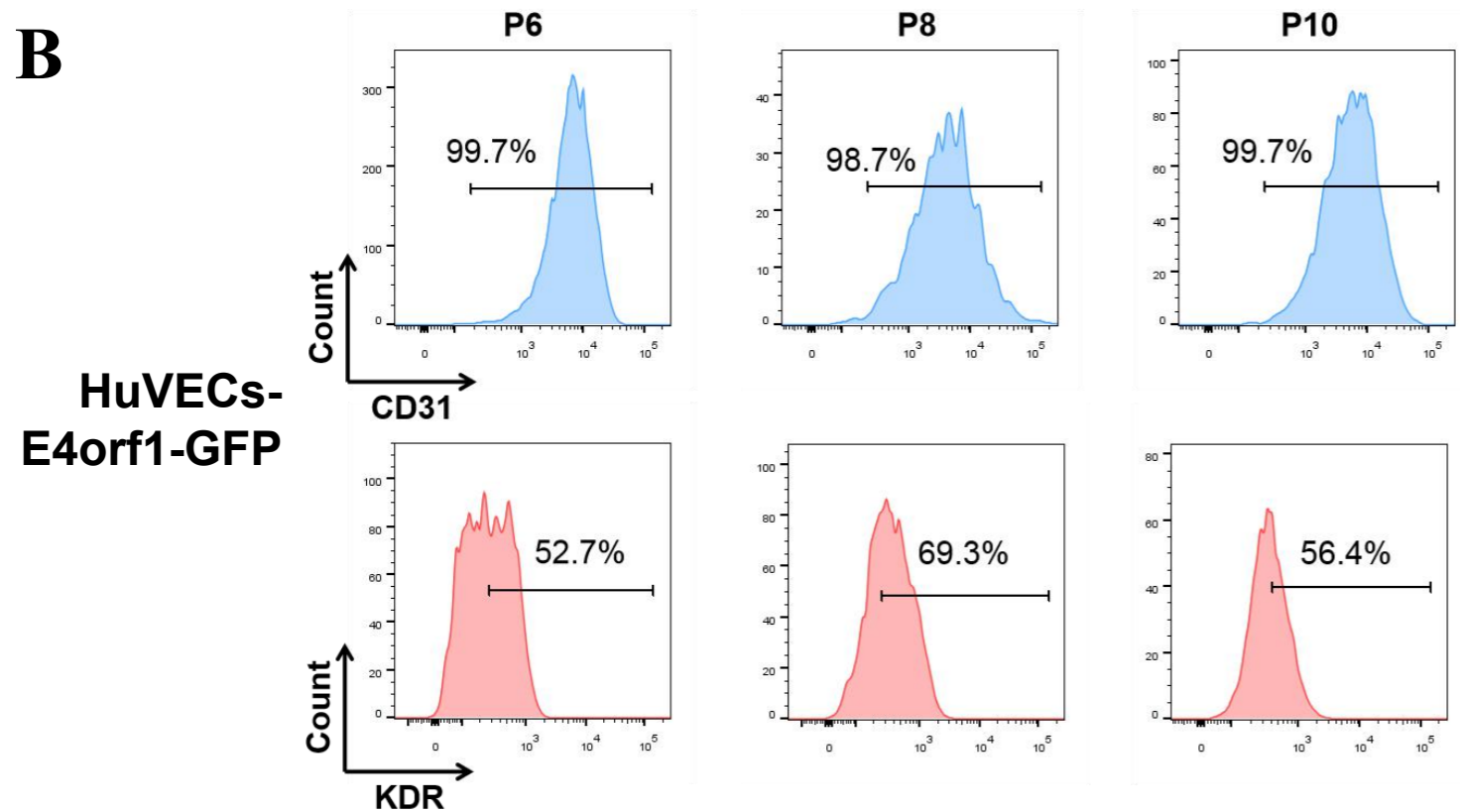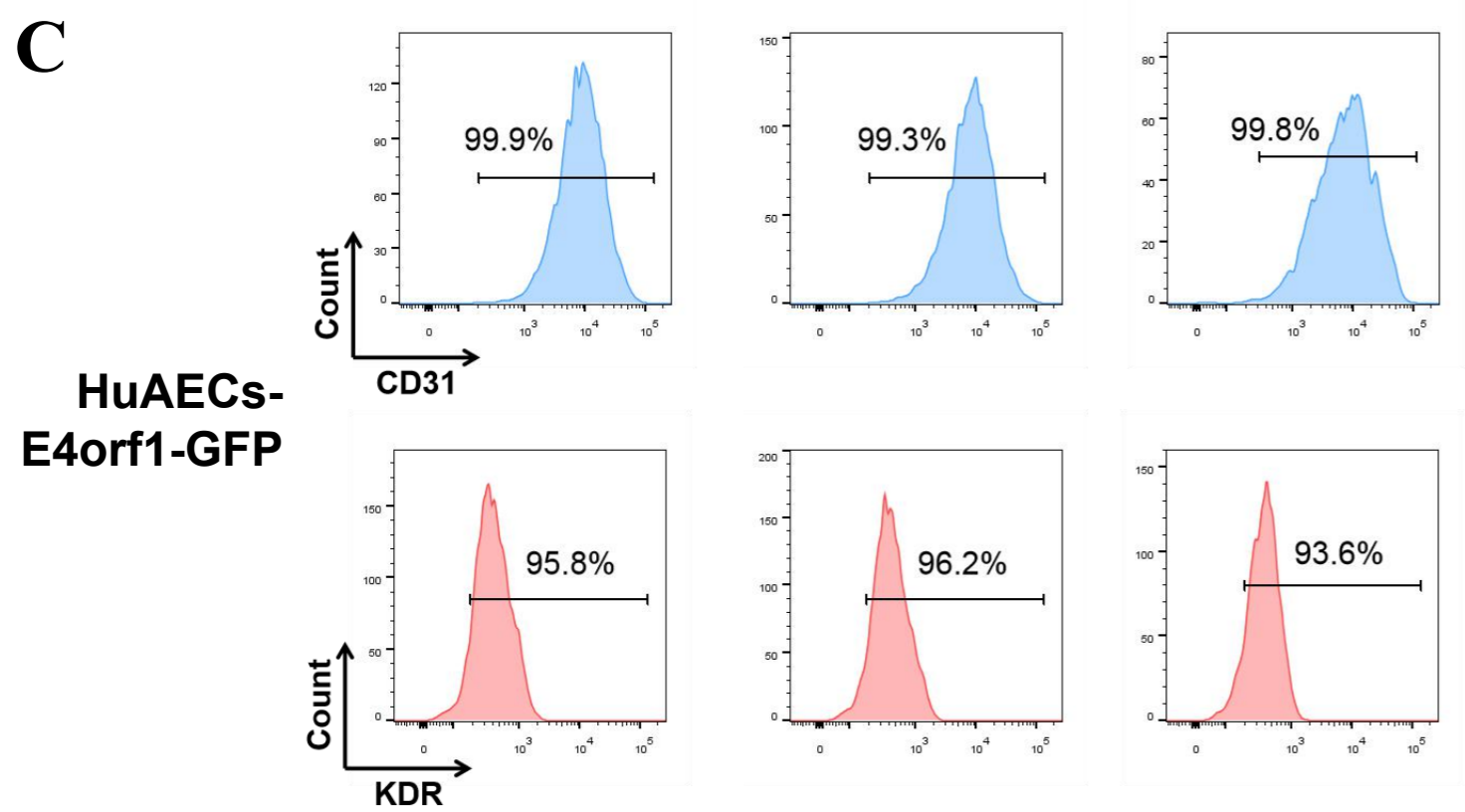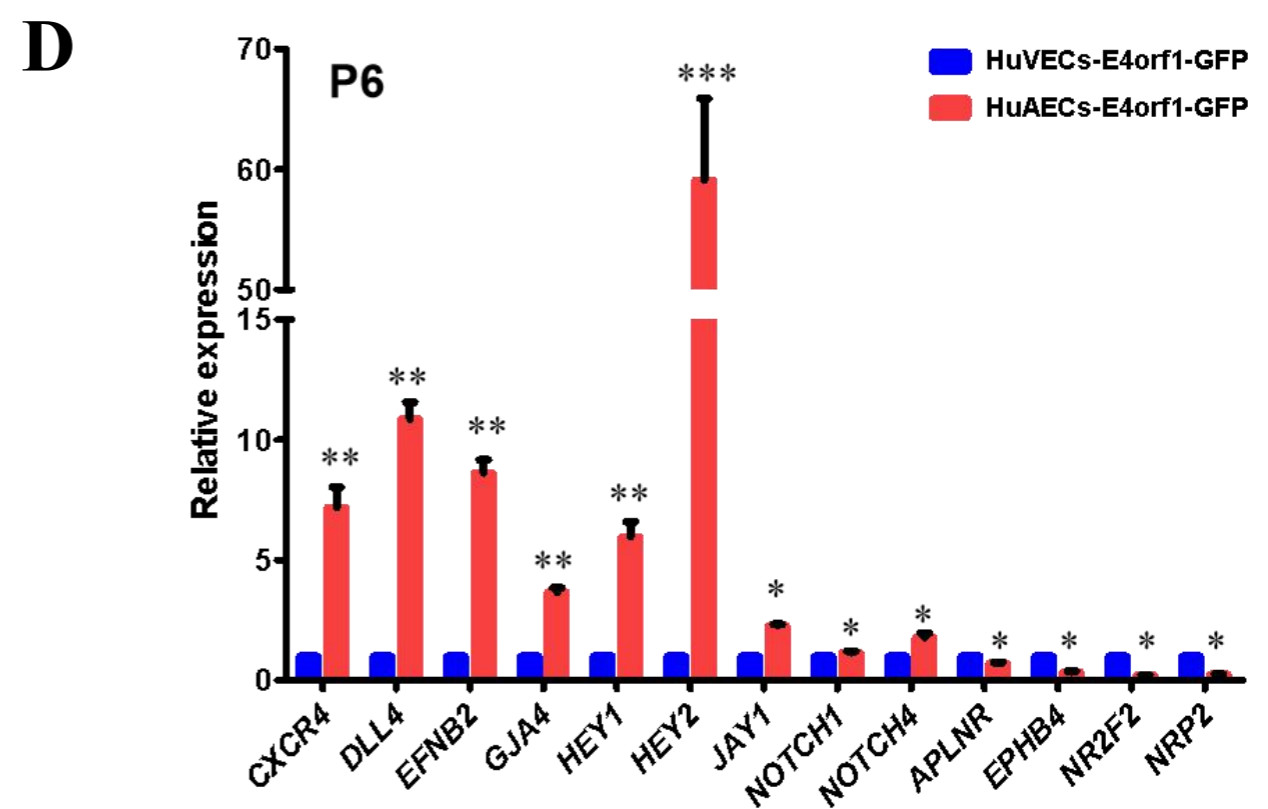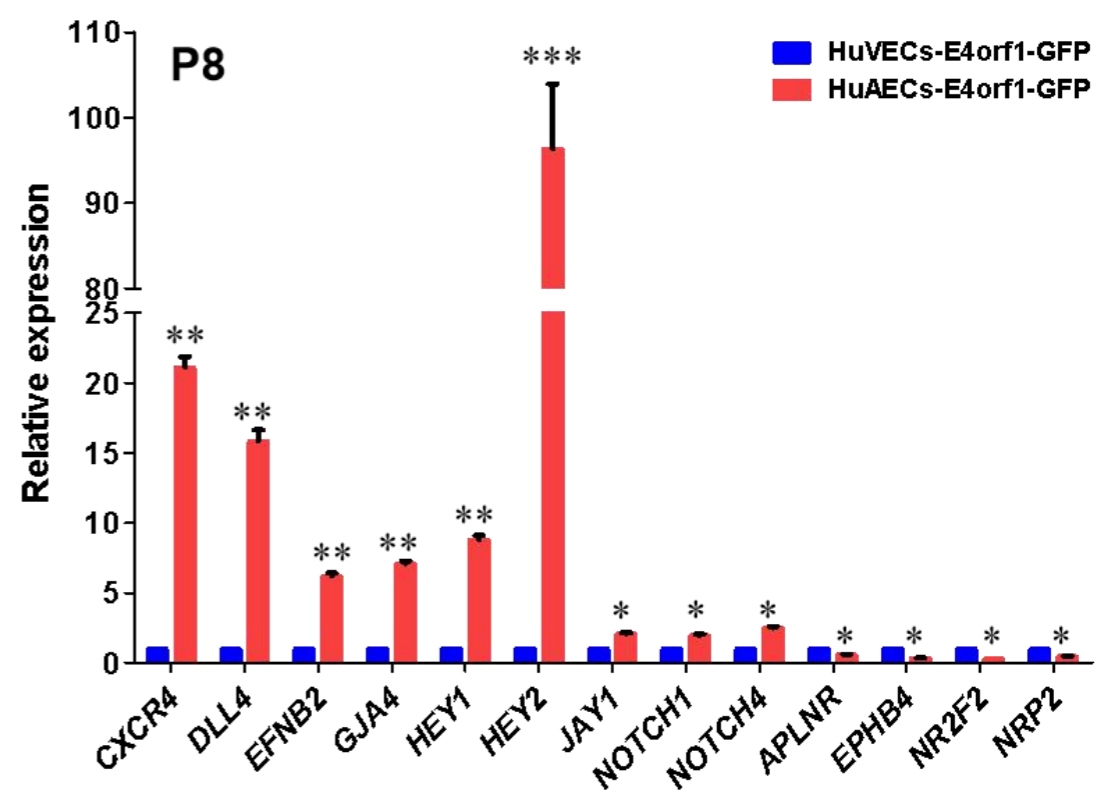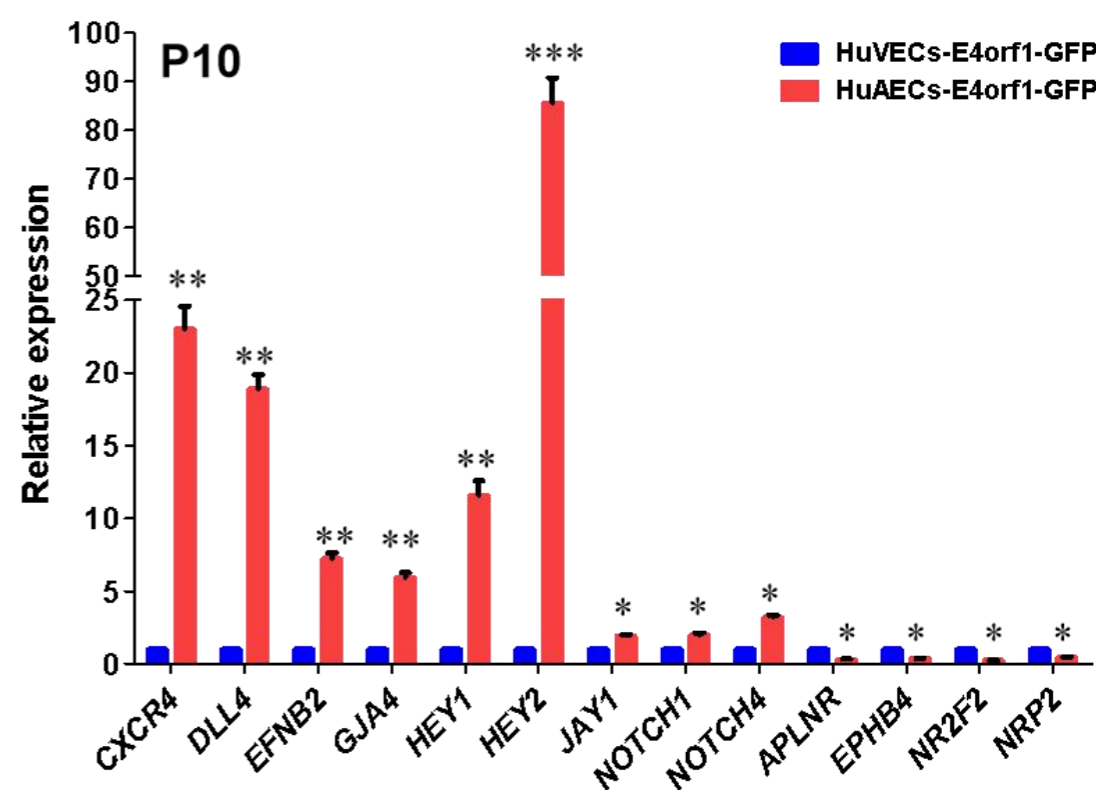

Supplement: Supplementary file 1 — Additional file 1: Supplementary Fig. 1. The endothelial cell phenotype retained within 10 passages after transfection. (A) vWF staining negative control. (B) Flow cytometric analysis of CD31 and KDR expression in HuVECs-E4orf1-GFP. (C) Flow cytometric analysis of CD31 and KDR expression in HuAECs-E4orf1-GFP. (D) qRT-PCR analysis of arterial and venous markers expression in HuVECs-E4orf1-GFP and HuAECs-E4orf1-GFP for different generations. *P < 0.05, **P < 0.01, ***P < 0.001; n = 3; Scale bar: 200 μm. [file 13287_2020_1880_MOESM1_ESM.pdf]

# Supplementary Figure 2

A

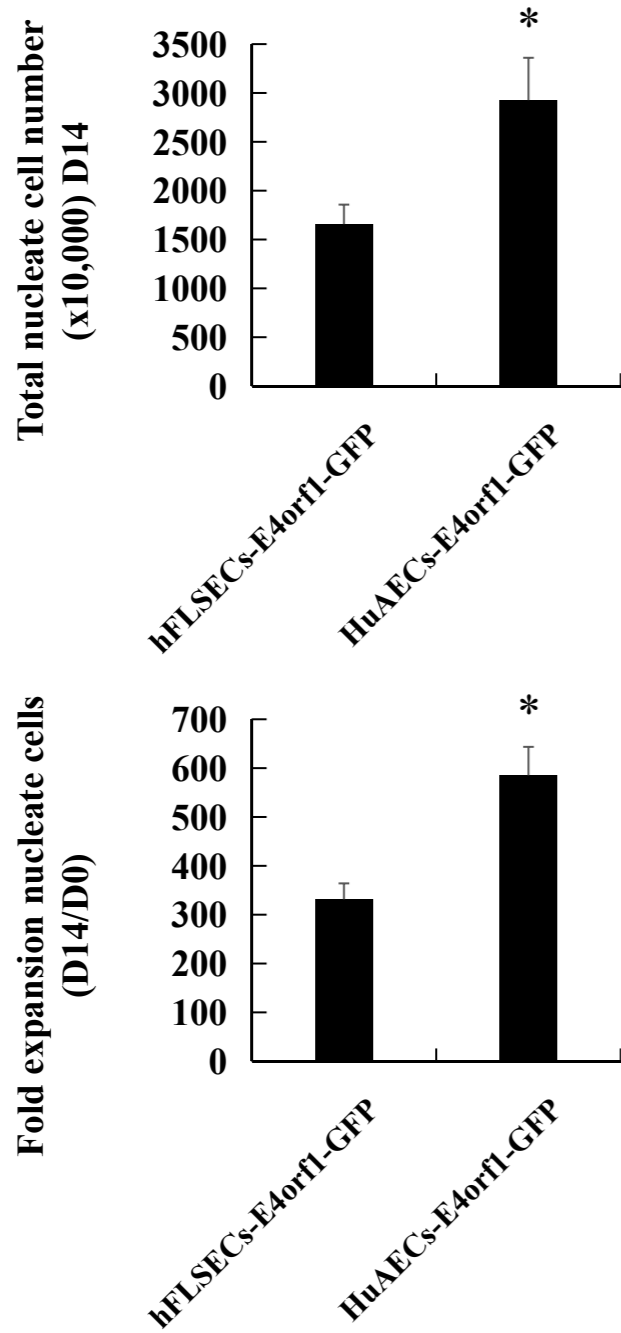

C

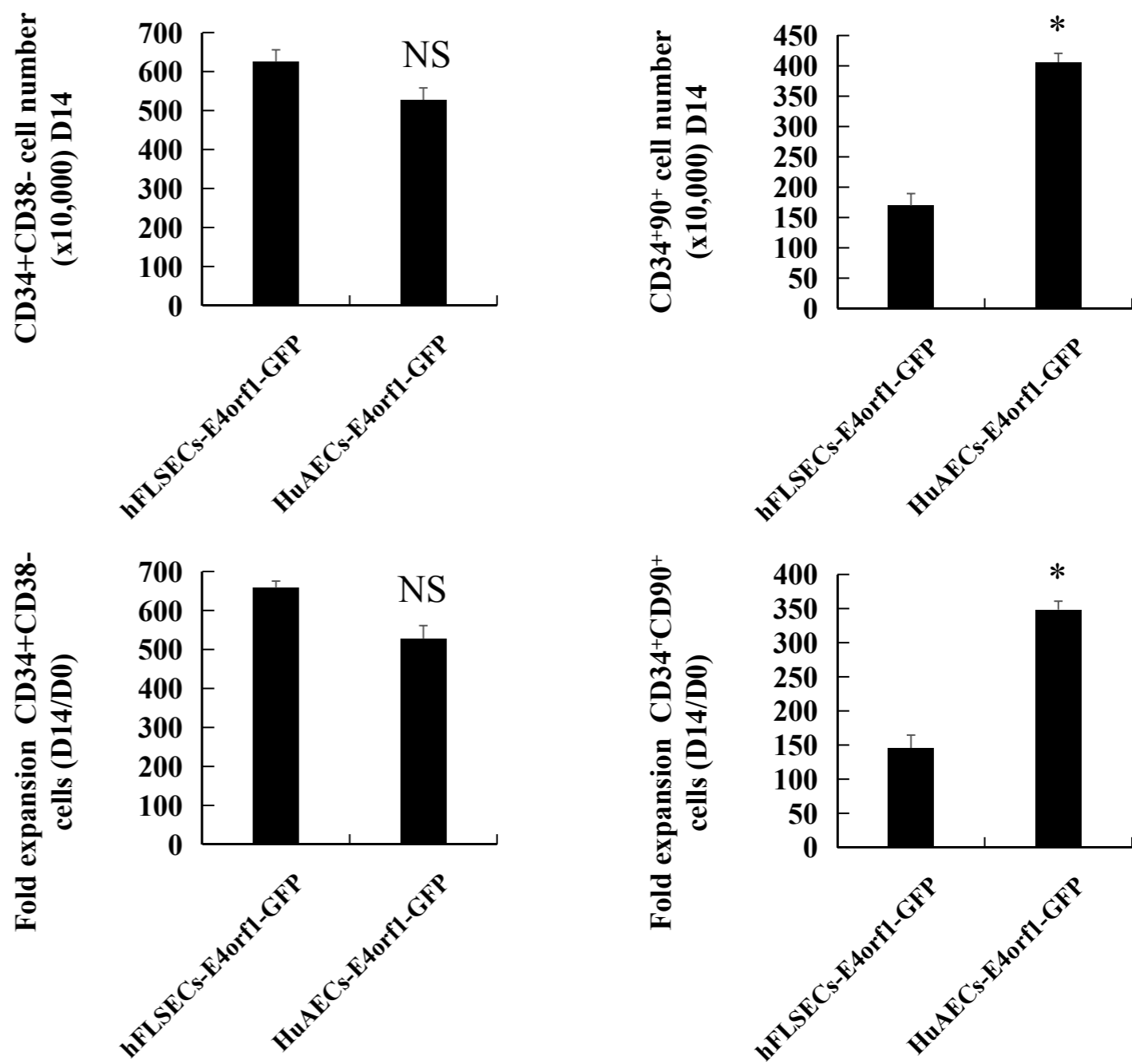

B

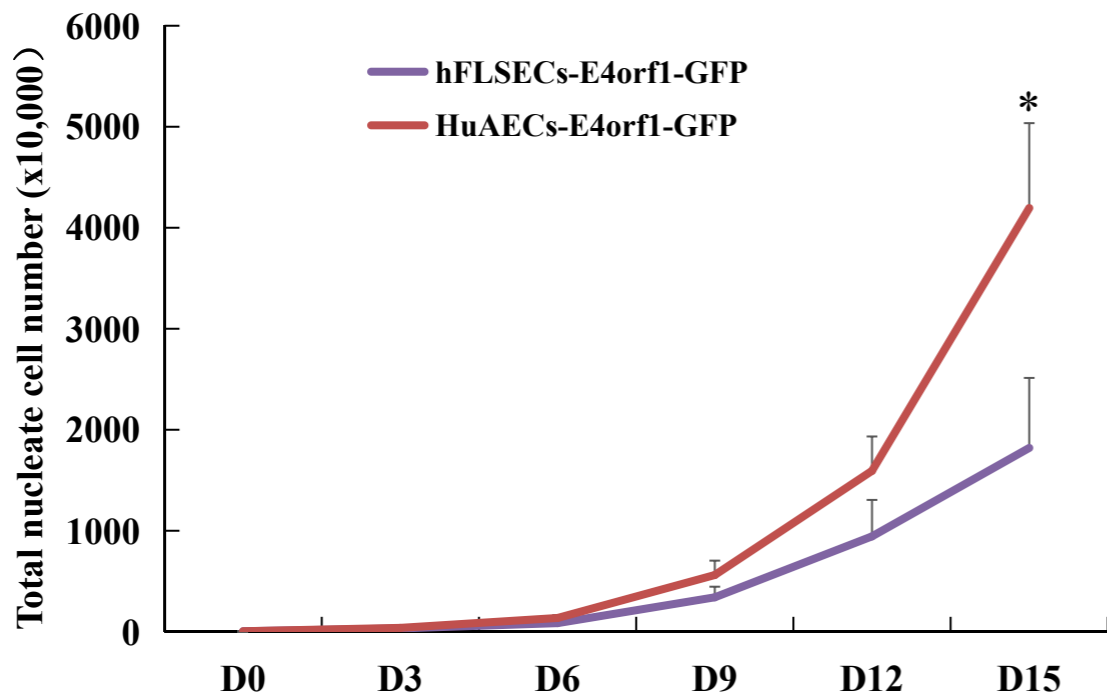

D

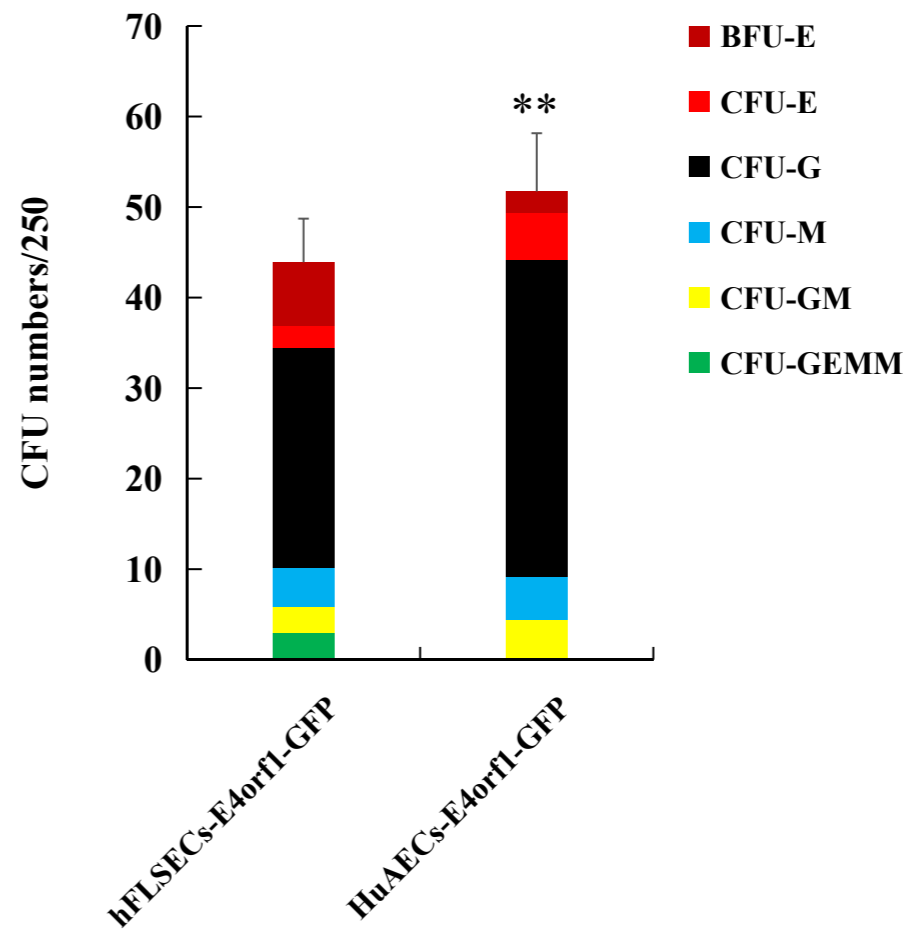

Supplement: Supplementary file 2 — Additional file 2: Supplementary Fig. 2. Contrasting the effect of HuAECs-E4orf1-GFP and hFLSECs-E4orf1-GFP on CD34+ hCB cell expansion. (A) TNC expansion at day 14. (B) The cumulative curve of TNC in ex vivo expansion. (C) CD34+CD38− (left panel) and CD34+CD90+ cell (right panel) expansion. (D) CFU number of amplified CD34+ hCB cells in HuAECs-E4orf1-GFP coculture or hFLSECs-E4orf1-GFP coculture. NS means ‘no significant difference’, *P < 0.05, **P < 0.01; n = 5. [file 13287_2020_1880_MOESM2_ESM.pdf]

# Supplementary Figure 3

A

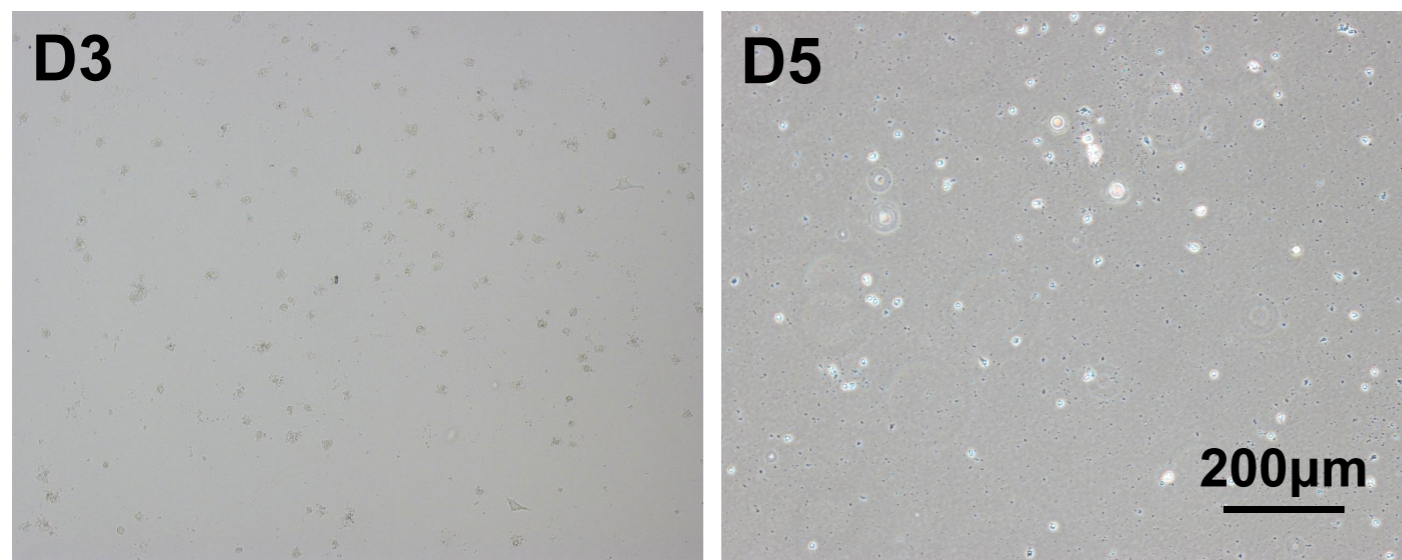

C

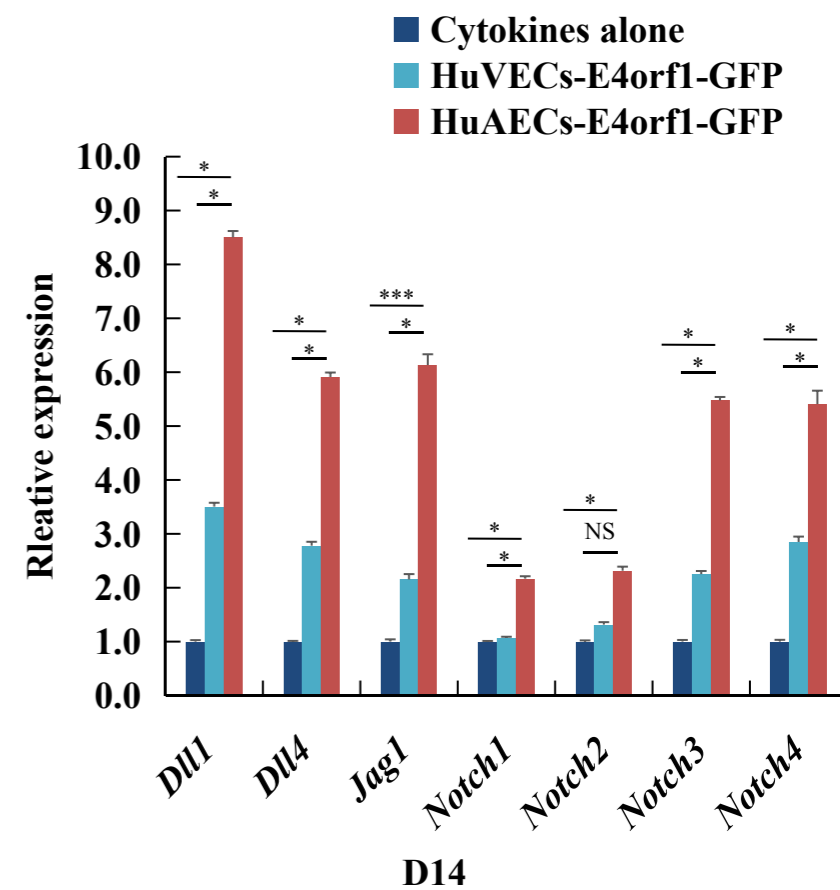

D

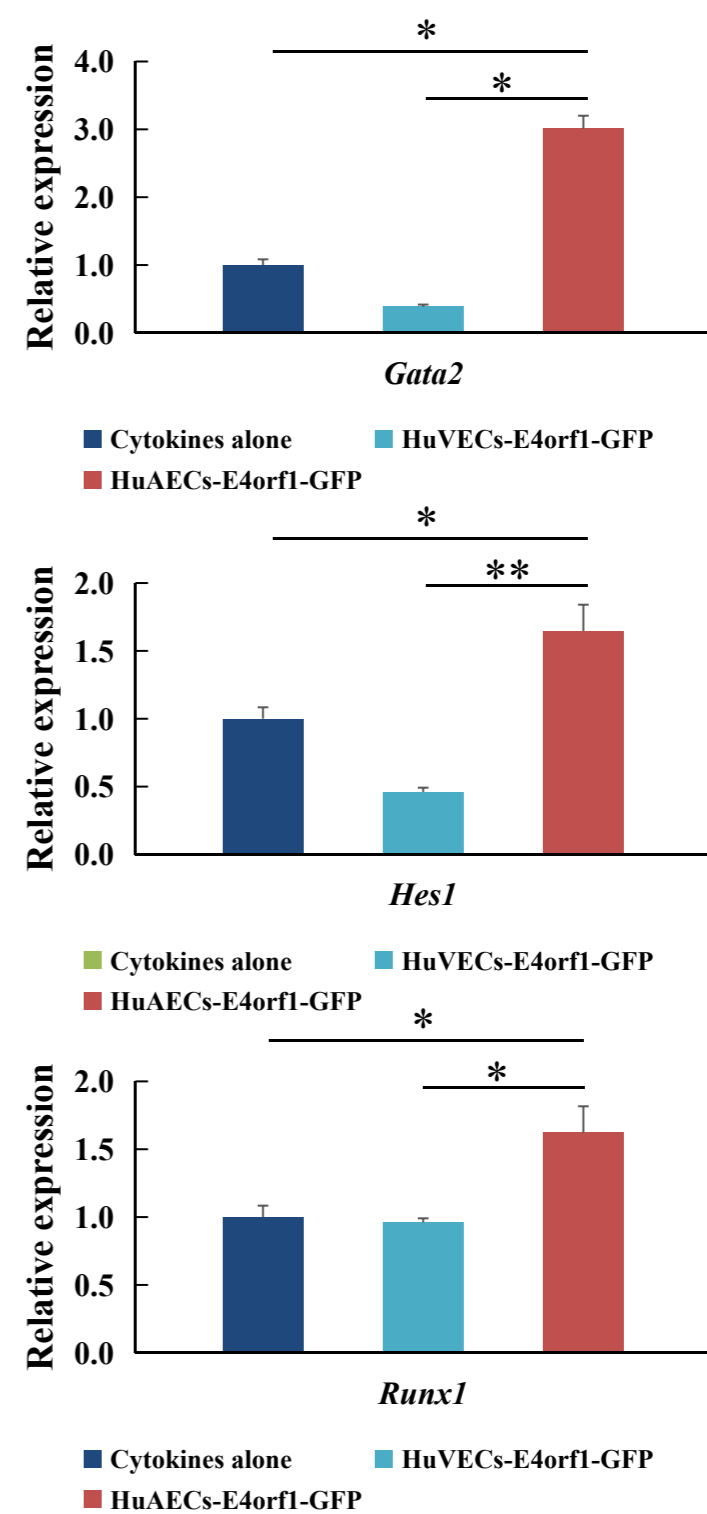

B

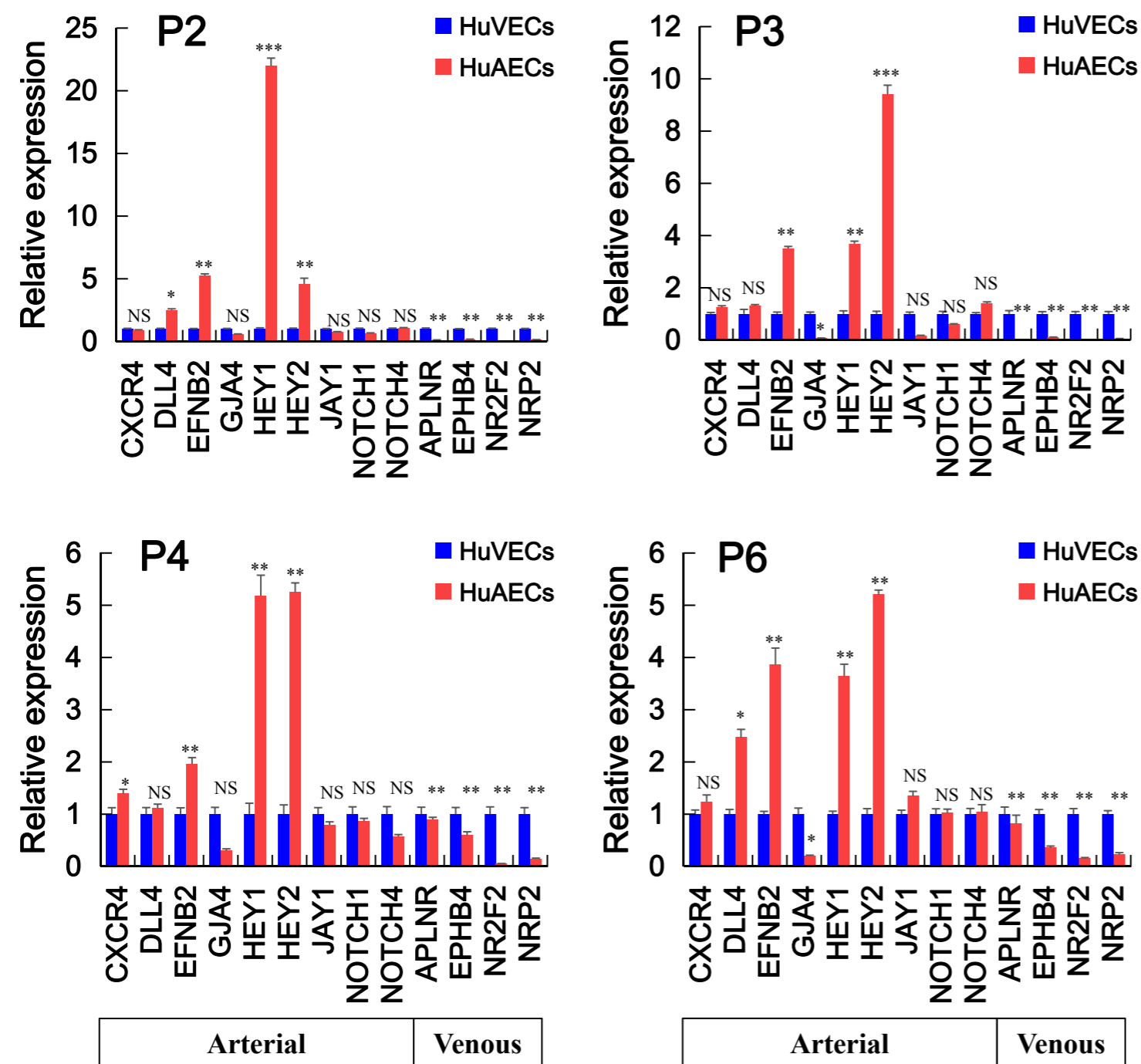

Supplement: Supplementary file 3 — Additional file 3: Supplementary Fig. 3. Gene expression of PECs and expanded CD34+ hCB cells. (A) The morphology of PECs cultured in serum-free, cytokine-free condition. Scale bar: 200 μm. (B) qRT-PCR analysis of arterial and venous markers expression in primary HuVECs and HuAECs for different generations. (C) Comparison of the relative transcript levels of Notch ligands and receptors in expanded CD34+ hCB cells from different groups at day 14. (D) The transcript levels of Notch target genes (GATA2, HES1 and RUNX1) in expanded CD34+ hCB cells at day 14. NS means ‘no significant difference’, *P < 0.05, **P < 0.01, ***P < 0.001; n = 3. [file 13287_2020_1880_MOESM3_ESM.pdf]

# Supplementary Figure 4

**A**

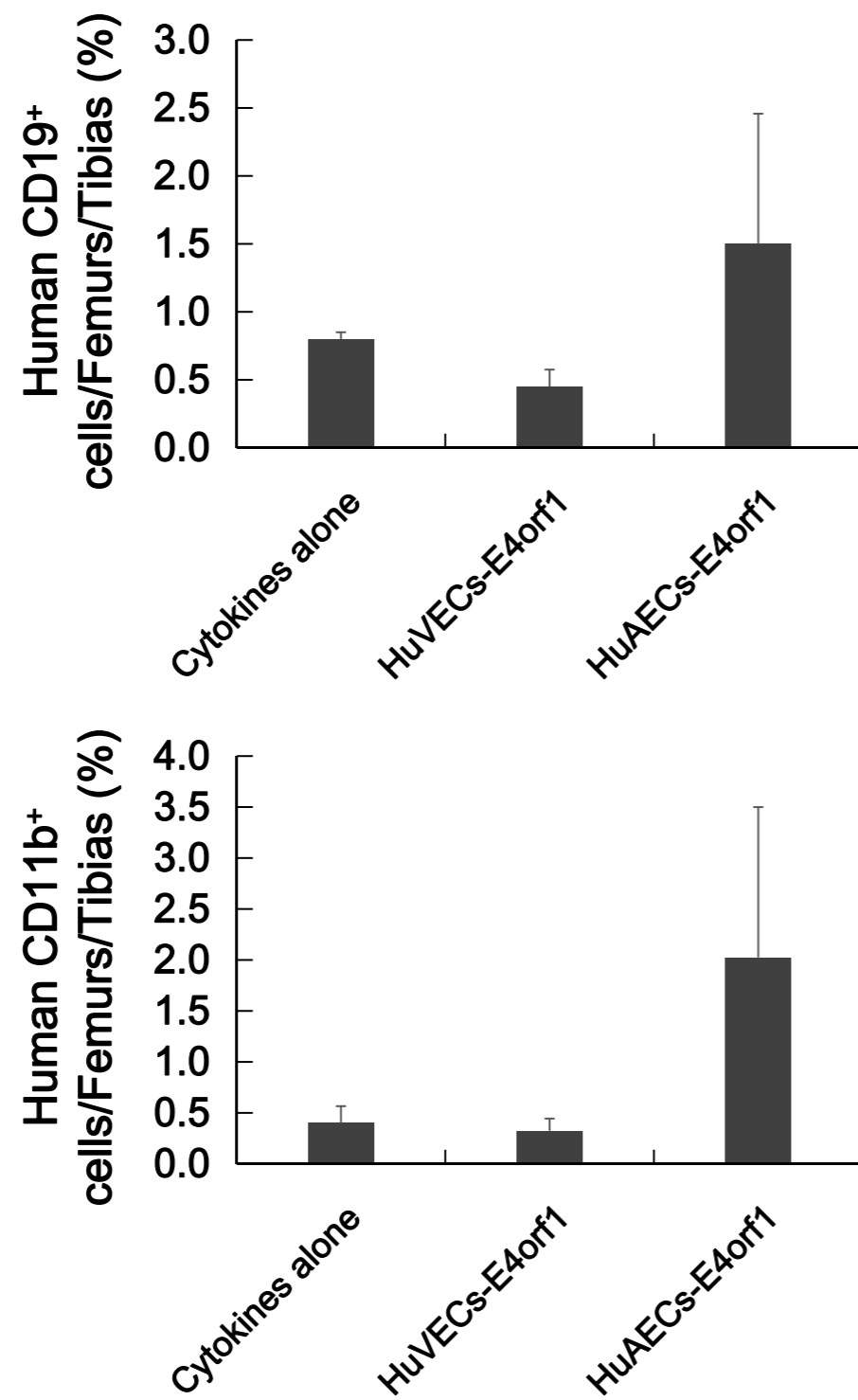

**B**

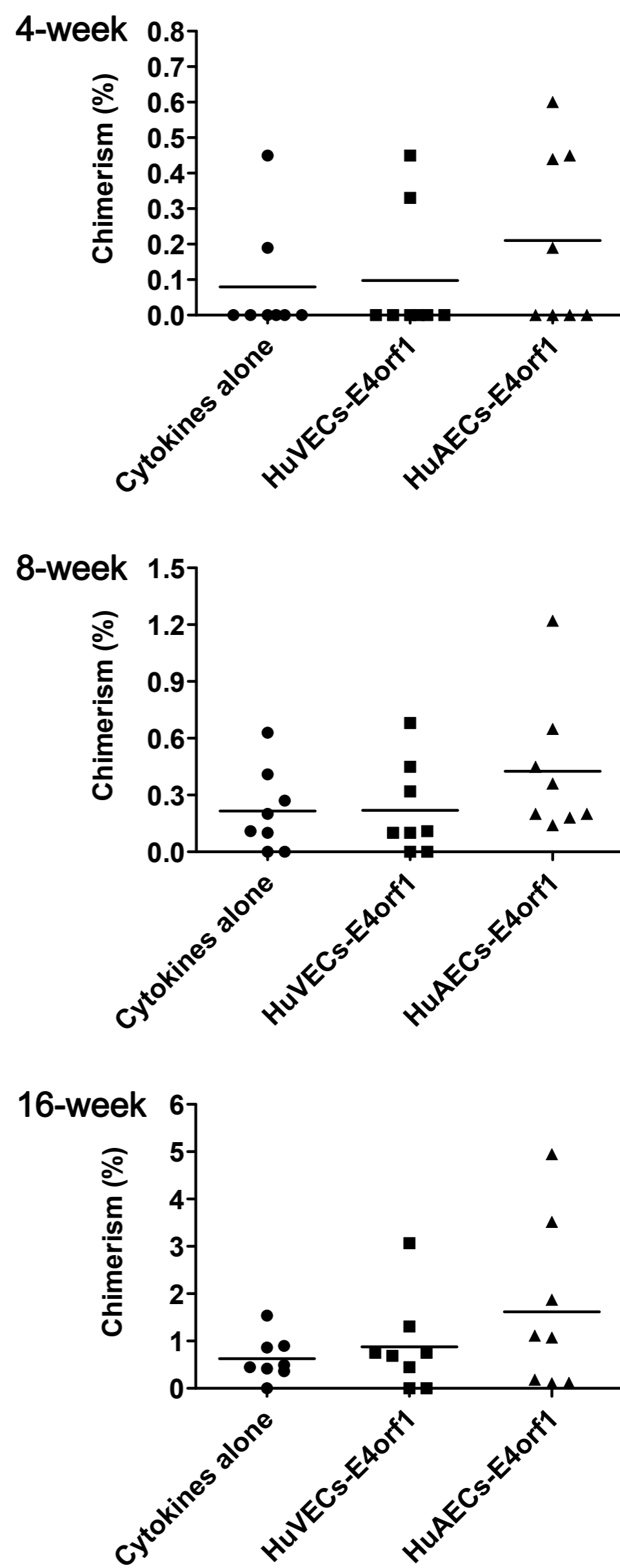

Supplement: Supplementary file 4 — Additional file 4: Supplementary Fig. 4. Multilineage engraftment measurements following transplantation. (A) Multilineage human hematopoietic cell engraftment after 16-week transplantation. (B) Percentage of engrafted human CD45+ hematopoietic cells at 4, 8 and 16 weeks after transplantation. n = 8. [file 13287_2020_1880_MOESM4_ESM.pdf]
